# Lung Postmortem Autopsy Revealing Extramedullary Involvement in Multiple Myeloma Causing Acute Respiratory Distress Syndrome

**DOI:** 10.1155/2014/635237

**Published:** 2014-08-06

**Authors:** Aurélie Ravinet, Sébastien Perbet, Romain Guièze, Richard Lemal, Renaud Guérin, Guillaume Gayraud, Jugurtha Aliane, Aymeric Tremblay, Julien Pascal, Albane Ledoux, Carine Chaleteix, Pierre Dechelotte, Jacques-Olivier Bay, Jean-Etienne Bazin, Jean-Michel Constantin

**Affiliations:** ^1^Réanimation Adultes et Unité de Soins Continus, CHU Estaing, 1 Place Lucie-et-Raymond-Aubrac, CHU Clermont-Ferrand, 63003 Clermont-Ferrand, France; ^2^Service d'Hématologie, CHU Estaing, CHU Clermont-Ferrand, 1 Place Lucie-et-Raymond-Aubrac, 63003 Clermont-Ferrand, France; ^3^R2D2, EA 7281, INSERM, Faculté de Médecine, Université d'Auvergne, Place Henri Dunant, 63001 Clermont-Ferrand, France; ^4^INSERM CIC-501, EA 7283, CHU Estaing, Université d'Auvergne, 1 Place Lucie-et-Raymond-Aubrac, 63003 Clermont-Ferrand, France; ^5^Service d'Anatomo-Pathologie, CHU Estaing, CHU Clermont-Ferrand, 1 Place Lucie-et-Raymond-Aubrac, 63003 Clermont-Ferrand, France

## Abstract

Pulmonary involvement with multiple myeloma is rare. We report the case of a 61-year-old man with past medical history of chronic respiratory failure with emphysema, and a known multiple myeloma (Durie and Salmon stage III B and t(4;14) translocation). Six months after diagnosis and first line of treatment, he presented acute dyspnea with interstitial lung disease. Computed tomography showed severe bullous emphysema and diffuse, patchy, multifocal infiltrations bilaterally with nodular character, small bilateral pleural effusions, mediastinal lymphadenopathy, and a known lytic lesion of the 12th vertebra. He was treated with piperacillin-tazobactam, amikacin, oseltamivir, and methylprednisolone. Finally, outcome was unfavourable. Postmortem analysis revealed diffuse and nodular infracentimetric infiltration of the lung parenchyma by neoplastic plasma cells. Physicians should be aware that acute respiratory distress syndrome not responding to treatment of common causes could be a manifestation of the disease, even with negative BAL or biopsy and could be promptly treated with salvage therapy.

## 1. Introduction

Multiple myeloma is a hematological malignancy characterized by the occurrence of plasma cell tumor within the bone marrow and less common extramedullary plasmocytomas [1]. Pulmonary involvement with multiple myeloma is rare and may be difficult to distinguish from more common interstitial diseases in case of acute respiratory failure.

## 2. Case Report

A 61-year-old man was diagnosed with an immunoglobulin G (IgG) kappa multiple myeloma, Durie and Salmon stage III B, and t(4; 14) translocation. His past medical history included active smoking and chronic respiratory failure with emphysema. He was treated by bortezomib-thalidomide-dexamethasone (BTD) and then autologous stem cell transplantation conditioned by melphalan 200 mg/m^2^ 4 months later. He had a partial response and then received 2 cycles of BTD as consolidation therapy. He presented progressive disease 6 months later with anemia, thrombocytopenia, and increase of serum M-protein (IgG *κ*) of 28 g/L (normal < 12 g/L).

He was then admitted for recurrent fever, cough, and dyspnea. Laboratory data revealed a positive influenza B sample. Routine blood investigation showed the following results: hemoglobin (8.9 g/dL), total leukocyte count of 6.510/mm^3^ with neutrophils (68%), lymphocytes (8%), monocytes (8%), metamyelocytes (3%), myelocytes (6%) and plasma cells (7%), and platelet count of 12,000/mm^3^. The chest radiograph showed bilateral multifocal areas of nodular infiltration. High-resolution computed tomography of the thorax showed severe bullous emphysema and diffuse, patchy, multifocal air space infiltration bilaterally with nodular character, small bilateral pleural effusions, mediastinal lymphadenopathy, and a known lytic lesion of the 12th vertebra. Computed tomography of the brain did not reveal argument for brain injury or involvement. He was first treated by piperacillin-tazobactam, amikacin, oseltamivir, and methylprednisolone 1 mg/kg/day for 12 days.

**Figure 1:**
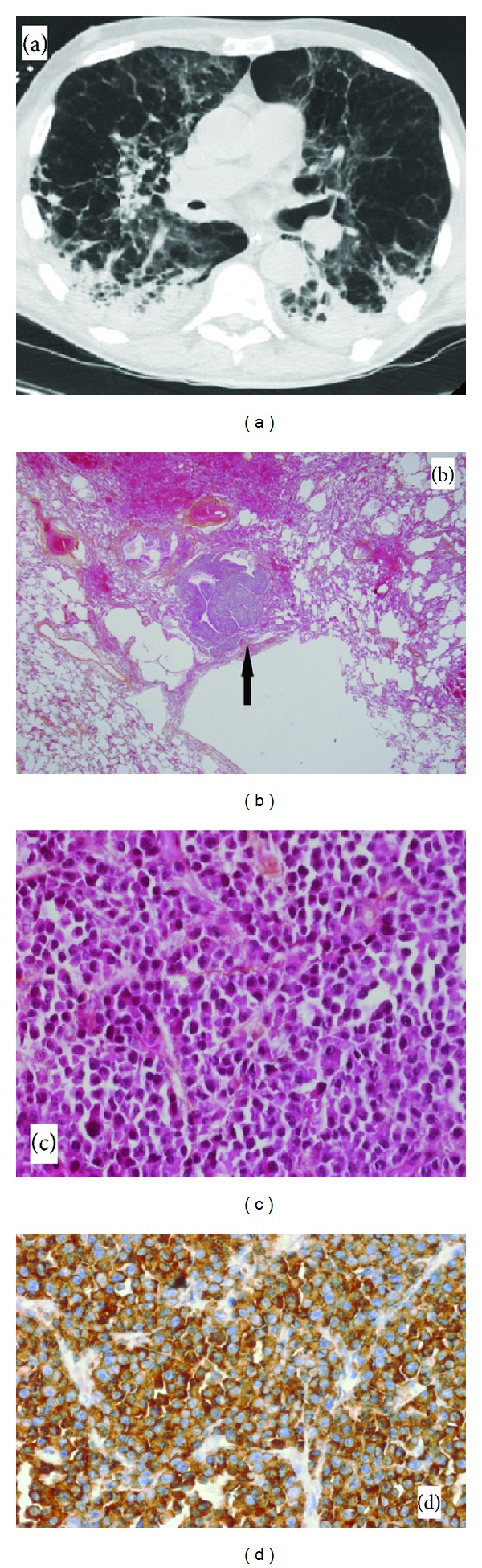
(a) Computed tomography thorax revealed severe bullous emphysema and diffuse, patchy, multifocal air space infiltration bilaterally with a nodular character, small bilateral pleural effusions, and mediastinal lymphadenopathy. (b) Lung tissue specimen from the autopsy revealing nodular tumoral infiltrate (hematoxylin and eosin ×2.5). (c) Lung tissue specimen from the autopsy revealing characteristic abnormal plasma cell infiltrates (hematoxylin and eosin ×40). (d) Immunohistochemical staining of the tissue specimen showing multiple myeloma cell positive for IgG (original ×40).

The patient's respiratory status quickly declined and he was admitted to the ICU requiring intubation and mechanical ventilation. Bronchoalveolar lavage (BAL) yielded fluid did not reveal malignant cells or pathogens. He presented a delirium and agitation syndrome. The cerebrospinal fluid (CSF) examination showed an IgG count of 354 mg/mL (normal < 35 mg/mL), proteinorachia of 1.23 g/L, glycorrhachia of 3.9 mmol/L, 700 red blood cells, and 2 normal white blood cells per microliter without plasma cells. He received other antimicrobial therapies for pneumonia and methylprednisolone for myeloma. He was judged to be a poor candidate for more aggressive salvage therapy taking into account the poor prognosis associated with t(4; 14) translocation and the progressive multiorgan failure. In keeping with the patient's advance directive and after consultation with his family, supportive care was withdrawn. Autopsy revealed diffuse and nodular infra-centimetric infiltration of the lung parenchyma by neoplastic plasma cells. Immunohistochemistry confirmed the pulmonary infiltration of monotypic plasma cells in the lung biopsy (Figure 1).

## 3. Discussion

Extramedullary dissemination of multiple myeloma occurs in advanced disease, but it is rare. The sites of extramedullary dissemination are spleen, liver, lymph nodes, kidney, thyroid or adrenal glands, testes, ovary, pericardium, intestinal tract, and skin, while lung involvement is extremely rare.

The reported thoracic manifestation of multiple myeloma is bony involvement of the thoracic cage or mediastinal lymphadenopathy [2]. The intraparenchymal causes of respiratory failure from multiple myeloma include alveolar septal amyloidosis, plasma cell infiltration of lung parenchyma, accumulation of alveolar paraproteins, and metastatic calcification of the alveolar walls and blood vessels [3–6].

Diagnosis of pulmonary parenchymal involvement by myeloma cells remains difficult. Only isolated cases with histological proofs have been reported in the literature. In a series of 958 patients with multiple myeloma, 10% had pulmonary findings, but only 4 cases were suggestive of plasma cell involvement and only one case was documented histologically [1]. The diagnosis of pulmonary infiltration can be sometimes established from BAL fluid demonstrating the presence of plasma cells [7, 8] or lung biopsy showing plasma cells infiltration in the interstitium [9, 10]. Our patient presented pulmonary disease with noncontributory BAL, no specific lesion as pulmonary infiltrate was detected by chest CT scan, and a lung biopsy was dangerous because of severe bullous emphysema. Involvement of the central nervous system was not retained with absence of abnormalities in computed tomography scan of the brain, of malignant plasma cells in the CSF and of plasma cell leukaemia (<20% malignant plasma cells) [11]. Elevation of IgG in the CSF could be reliable to a hemorrhagic puncture and in any case, cytological evidence cannot be replaced by the detection of a monoclonal M-component in the CSF, as changes in the blood-barrier allow immunoglobulins to pass into the CSF [12].

A greater clinical suspicion could conceivably have resulted in initiating more effective myeloma drugs than methylprednisolone. However multiple myeloma refractory to both proteasome inhibitors and immunomodulatory agents (IMiDs; double-refractory myeloma) has a poor prognosis with a median overall survival and progression-free survival of 9 months and 5 months, respectively [13]. While there is no standard treatment for these patients, several promising agents are under investigation. Notably, second-generation proteasome inhibitors (as carfilzomib) and third-generation IMiD (as pomalidomide, recently approved in France in August 2013) may be of interest for these patients.

Lung involvement occurs in case of progressive disease in the seventh decade [10, 14, 15] and must be suspected in case of uncontrolled respiratory failure after adequate antimicrobial therapy.

## 4. Conclusion

In case of multiple myeloma, physicians should be aware that acute respiratory distress syndrome not responding to treatment of common causes could be a manifestation of the disease, even with negative BAL or biopsy. It must be proven by adequate BAL or biopsy and be promptly treated with salvage therapy.
